# Genetic analysis of indel markers in three loci associated with Parkinson's disease

**DOI:** 10.1371/journal.pone.0184269

**Published:** 2017-09-05

**Authors:** Zhixin Huo, Xiaoguang Luo, Xiaoni Zhan, Qiaohong Chu, Qin Xu, Jun Yao, Hao Pang

**Affiliations:** 1 Department of Forensic Genetics and Biology, China Medical University, Shenyang North New Area, Shenyang, P.R., China; 2 Department of Neurology, 1st Affiliated Hospital of China Medical University, Shenyang, P.R., China; University of Iceland, ICELAND

## Abstract

The causal mutations and genetic polymorphisms associated with susceptibility to Parkinson’s disease (PD) have been extensively described. To explore the potential contribution of insertion (I)/deletion (D) polymorphisms (indels) to the risk of PD in a Chinese population, we performed genetic analyses of indel loci in *ACE*, *DJ-1*, and *GIGYF2* genes. Genomic DNA was extracted from venous blood of 348 PD patients and 325 age- and sex-matched controls without neurodegenerative disease. Genotyping of the indel loci was performed by fragment length analysis after PCR and DNA sequencing. Our results showed a statistically significant association for both allele *X* (alleles without *5*) vs. *5* (odds ratio = 1.378, 95% confidence interval = 1.112–1.708, *P* = 0.003) and genotype *5/X+X/X* vs. *5/5* (odds ratio = 1.681, 95% confidence interval = 1.174–2.407, *P* = 0.004) in the *GIGYF2* locus; however, no significant differences were detected for the *ACE* and *DJ-1* indels. After stratification by gender, no significant differences were observed in any indels. These results indicate that the *GIGYF2* indel may be associated with increased risk of PD in northern China.

## Introduction

Parkinson’s disease (PD) is the most common neurodegenerative disorder besides Alzheimer disease (AD) [1]. Due to the presence of Lewy bodies (LBs) and loss of dopaminergic neurons in the substantia nigra of the midbrain, the clinical symptoms of PD are mainly characterized by tremors at rest, rigidity, bradykinesia, and posture disorders [2,3]. The etiology of slowly progressive parkinsonian syndromes remains elusive, although certain genetic and environmental factors have been shown to contribute to PD [3].

Over the last decade, research has shed light on the relationship between some candidate genetic markers and PD in different populations [4–8]. These genetic markers include single nucleotide polymorphisms (SNPs), which occupy the majority of PD genetic investigations; rearrangements; and insertion (I)/deletion (D) polymorphisms (indels). However, very few indels have been evaluated in PD studies, especially in Chinese individuals. Some of these indels are located in functionally important sites of human genes and can, therefore, potentially influence pathogenicity [9]. Additionally, some indel polymorphisms that are present in non-functional regions are susceptible to disease and may also be linked to other pathogenic markers. Therefore, we analyzed the association between indels and PD risk in the current study.

Angiotensin converting enzyme (ACE), a membrane-bound zinc metallopeptidase, regulates blood pressure and blood volume by converting angiotensin I to angiotensin II and by degrading bradykinin. Previous studies have suggested that it may be involved in the pathogenesis of neurodegenerative disorders such as PD, AD, Huntington’s disease, and autism [10,11]. Furthermore, the primary loss of substance P due to ACE hydrolysis has been shown to be a contributing factor in the pathogenesis of PD [12–14]. The polymorphism in the *ACE* gene is an indel of the 287-bp *Alu* repetitive sequence in intron 16 (rs1799752), which leads to a deletion allele and insertion allele [15].

The *DJ-1* (*PARK7*) gene is located on chromosome 1p36 [16], the product of which is a peptidase C56 family protein. It has been reported that oxidative stress is involved in the pathology of neurodegenerative diseases, such as PD and AD [17]. Interestingly, the DJ-1 protein protects dopaminergic neurons from oxidative stress [18,19]. In 2003, Vincenzo Bonifati *et al*. reported that *DJ-1* gene mutations are associated with patients in autosomal recessive early onset Parkinsonism [19]. Likewise, the *DJ-1* gene with an 18-bp indel polymorphism (g.168_185del; rs864309640; ss1966650459) in its 5’ UTR has been shown to increase the risk of PD [20,21].

Grb10 interacting GYF Protein 2 (GIGYF2), which cooperates with the Grb10 adapter protein to stimulate IGF-1 receptors [22], was identified in dopaminergic neurons in the substantia nigra pars compacta (SNpc) of PD brain tissue and is involved in the regulation of tyrosine kinase receptor signaling at endosomes [23]. The *GIGYF2* gene was reported to be a susceptibility gene- that is accountable for PARK11-linked PD [24] and is located in the chromosomal region 2q36-37 [25]. Lautier *et al*. [24] described all *GIGYF2* coding exons through complete sequencing in Italian and French cohorts; their data strongly support that *GIGYF2* at the PARK 11 locus has a causal role in familial PD. Among them, one of the mutations showed a trinucleotide indel in exon 29 of the *GIGYF2* gene (NG_011847), which is described as being encoded in exon 25 of *GIGYF2* in most other published reports.

Three indel polymorphisms in *ACE*, *DJ-1* and *GIGYF2* have been reported to be associated with, as potential susceptibility factors, PD and AD pathophysiology [12,14,26,27]. However, the results from association studies evaluating the relationship between the above indel markers and distribution of PD risk are still unclear. In a northern Chinese population, we investigated many point mutations associated with Parkinson's disease. Nevertheless, these were insufficient to explain the association with PD. Therefore, we investigated the frequency distributions of indels markers in three loci to explain the pathogenesis of PD in this population.

## Materials and methods

### Study population

A total of 348 ethnic Chinese individuals with idiopathic PD [mean age (standard deviation) = 63.80 (13.74) years; age at onset (standard deviation) = 56.96 (10.55)] from north China represented the patient group, including 183 male and 165 female patients. All patients were diagnosed by movement disorder neurologists from the First Affiliated Hospital of China Medical University in Liaoning province in China according to the British Parkinson’s Disease Society Brain Bank criteria for the clinical diagnosis of PD. The patients exhibited at least two of the three cardinal symptoms and signs for PD (tremor, rigidity, and bradykinesia) and also had a positive response to levodopa therapy. A total of 325 unrelated volunteers [mean age (standard deviation) = 66.87 (16.12) years] from the local community formed the control group, including 220 males and 105 females. The number of healthy female volunteers was limited in this investigation. Control participants were in good health and without a history of neurodegenerative disease. The scientific experimental protocol was approved by the ethics committee on Human Research of the China Medical University. All methods were performed in accordance with the guidelines of the Declaration of Helsinki. An informed consent agreement was signed by all individuals.

### Genomic DNA isolation and genotyping

Venous blood samples were obtained from the participants. Genomic DNA was extracted via the sodium dodecyl sulfate-proteinase K phenol-chloroform method.

The three indels were genotyped by fragment length analysis after PCR. The design of specific primers and related parameters is shown in Table 1. PCR was conducted in 20 μl volumes containing approximately 50 ng of genomic DNA, 2 × PowerTaq PCR MasterMix (BioTeke, Beijing, China) and 10 μM of each specific primer. PCR was carried out on a Thermo Cycler Block (Thermo Fisher Scientific, Vantaa, Finland) with initial denaturation at 94°C for 3 min, followed by 35 cycles of denaturing for 20 s at 94°C, annealing for 20 s (60°C for *ACE* and *GIGYF2*; 65°C for *DJ-1*), extension at 72°C (30 s for *ACE*; 15 s for *DJ-1*; 8 s for *GIGYF2*); and a final extension at 72°C for 1 min. PCR products were separated by electrophoresis on a 1% agarose gel for the *ACE* indel and on a 6% non-denaturing polyacrylamide gel for *DJ-1* and *GIGYF2* and were then visualized under a UV transilluminator after staining with ethidium bromide. All polymorphisms of *ACE*, *DJ-1*, and *GIGYF2* were analyzed in the 348 PD cases and 325 controls.

**Table 1:** The information of indel loci and PCR parameters.

Genes	Location in gene	Indel polymorphism	Primer direction	Sequence (5’→ 3’)	Allele size (base pairs)
*ACE*	Intron 16	287 bp	Forward	GGGGACTCTGTAAGCCACTG	532/245
			Reverse	TCCCATGCCCATAACAGGTC	
*DJ-1*	Intron 1	18 bp	Forward	GTGGGGTGAGTGGTACCCAA	248/230
			Reverse	GTCAGTCAAATCCAACGCCA	
*GIGYF2*	Exon 29	3 bp	Forward	TTCTGAGGCCAAGGAGTTTGC	159–192
			Reverse	ATCCTCAGAGGTACCGCATACA	

### Capillary electrophoresis for *GIGYF2*

To precisely determine the fragment length of the detected alleles in the *GIGYF2* locus, the forward primer for PCR amplification was labeled with a fluorescent dye, 6-carboxylfluorescein (6-FAM). PCR products with fluorescence were detected using capillary electrophoresis on an ABI Prism 3130 Genetic Analyzer (Applied Biosystems, USA) and analyzed using GeneMapper 2.1 analysis software. Additionally, the fluorescent ladder GeneScan^TM^ 600 Liz (Applied Biosystems, USA) was used as an internal size marker.

### DNA sequencing

To further confirm the sequences of all alleles containing the three indel polymorphisms, homozygous samples representing different alleles were first amplified and then directly sequenced. For heterozygous samples, mainly in the *GIGYF2* locus, we performed PCR amplification first. After PCR products were separated via a 6% polyacrylamide gel electrophoresis (PAGE) with 1 × TBE buffer and stained with ethidium bromide, the target fragments were excised from the gel and eluted with distilled water. The eluates were reamplified using the same primers and purified in Suprec-02 tubes (Takara, Shiga, Japan). The DNA sequences were determined using a Bigdye Terminator Cycle Sequencing Reaction Kit on an ABI Prism 3130 Genetic Analyzer (Applied Biosystems, USA).

### Statistical analysis

The Hardy-Weinberg equilibrium (HWE) test for frequency distribution was calculated using PowerMarker version 3.0 to detect whether the population was in Hardy-Weinberg equilibrium. For all genes, a mutant vs. wild-type model was used to test different distributions of genotype and alleles between patients and controls. To find the PD effect on gender we have divided our data into different groups (male PD cases vs. male controls, female PD cases vs. female controls, PD male vs. female cases and control male vs. female). We tested allele *5*, *6* and *7* against the rest of the alleles, but only allele *5* comparing with others showed the statistical significance. Therefore, in this study, *5* was treated as the major allele, and other allele classes (*X*) were merged to increase the statistics power for *GIGYF2*.

The *χ*2 test was used to test for association of the indel polymorphism and PD risk, using SPSS version 20.0 software. The statistical power to detect association of polymorphisms with PD exceeded 0.80 as estimated with PASS version 11.0 software. All analyses were carried out using a two-tailed *χ*2 test; *P* < 0.05 was considered as statistically significant. Bonferroni correction was utilized for multiple testing. Odds ratios (ORs), with their corresponding 95% confidence intervals (CIs), were used to evaluate the strength of the association between the polymorphisms and PD risk.

## Results

### Genotyping of three indel polymorphisms

**Fig 1:**
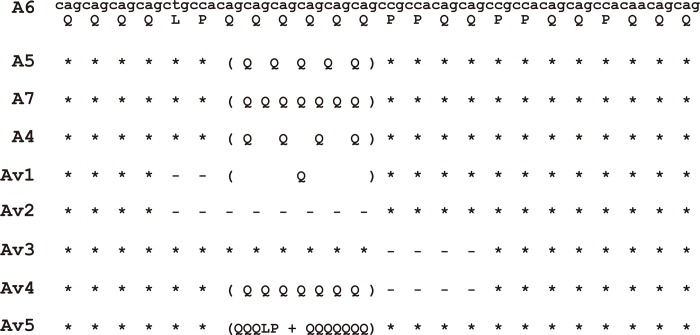
Nine allelic sequences were identified in the *GIGYF2* locus in this study. A6 (27.6%) is used as a reference sequence. Compared with A6, A5 (47.0%) and A7 (24.4%) were 3-bp repeated indels, and the others indels were different structural variations enclosing the poly-Q core unit (approximately 1.2%). The upper sequence represents base pairs of nucleic acids, and the lower sequence represents the corresponding amino acids.

The results showed that the frequency distribution for all indels did not deviate from Hardy-Weinberg equilibrium in each group (P > 0.05). The allele and genotype frequencies of the *ACE* and *DJ-1* loci and corresponding statistical power are shown in Table 2 and S1 Table. There were no statistically significant differences between PD patients and controls, as analyzed by the *χ*2 test (P > 0.05). For the indel polymorphism found presenting in exon 29 of the *GIGYF2* gene, more than three genotypes were observed, and all alleles were identified as a 3-bp repeat extension in the investigated samples, as indicated by both PAGE and capillary electrophoresis separation. To better understand the sequence architectures of these different alleles at the locus, we performed DNA sequencing. We found a total of nine sequence discrepancies. As shown in Fig 1, using allele 6 (A6) as a reference sequence [24], the indel polymorphism of the *GIGYF2* gene is more likely to be a short tandem repeat (STR) compared with alleles A5 and A7. Others indels exhibited a different structural variation enclosing the poly-Q core unit, which were identified as variant (*V*) alleles in our study.

**Table 2:** Allele frequency distribution and association analysis on indel loci of *ACE* and *DJ-1* genes.

Variables	PD Case (%)	Control (%)	*P*	OR (95% CI)	Power
*ACE*					
*I*^a^	458 (66)	414 (64)	0.418	1.097 (0.877, 1.372)	0.128
*D*	238 (34)	236 (36)			
*DJ-1*					
*D*^b^	66 (9)	62 (10)	0.972	0.994 (0.690, 1.430)	0.050
*I*	630 (91)	588 (90)			

a: variant allele in *ACE*

b: variant allele in *DJ-1*.

### The indel in the *GIGYF2* gene is associated with PD

For the complex indel located in exon 29 of *GIGYF2*, nine alleles and thirteen genotypes were identified, and the genotypic and allelic frequency distributions are shown in Table 3. The case-control analysis found a statistically significant difference after comparing the frequencies of all alleles (*P* < 0.05), but no difference was found after testing the frequencies of all genotypes. Considering that there are multiple alleles and genotypes present for this particular locus, we performed further tests of association for allelic and genotypic grouping analyses. Our results indicated that there was a significant difference between PD cases and controls when the *X* allele and *5/X+X/X* genotype of the indel in exon 29 of the *GIGYF2* gene was present compared with the *5* allele (OR = 1.378, 95% CI = 1.112–1.708, *P* = 0.003) and *5/5* genotype (OR = 1.681, 95% CI = 1.174–2.407, *P* = 0.004) (Table 4). Bonferroni correction was used in multiple testing, and the *P*-value was set at 0.025 (0.05/2) since two types of genetic models were used to analyze the association of the *GIGYF2* indel with PD. However, other allelic and genotypic grouping comparisons did not show any differences between PD cases and controls (S3 and S4 Tables).

**Table 3:** Frequency distribution and association analysis on indel locus of *GIGYF2* gene.

*GIGYF2*	PD Case (%)	Control (%)	*χ*2	*P*	Power
*5*	300 (0.431)	332 (0.511)	9.845	0.020	0.754
*6*	202 (0.290)	170 (0.262)			
*7*	185 (0.266)	144 (0.222)			
*V*	9 (0.013)	4 (0.006)			
*5/5*	67 (0.192)	93 (0.286)	11.959	0.153	0.683
*6/6*	25 (0.072)	23 (0.071)			
*7/7*	19 (0.055)	17 (0.052)			
*5/6*	83 (0.239)	79 (0.243)			
*5/7*	77 (0.221)	64 (0.197)			
*6/7*	68 (0.195)	45 (0.138)			
*5/V*	6 (0.017)	3 (0.009)			
*6/V*	1 (0.003)	0 (0.000)			
*7/V*	2 (0.006)	1 (0.003)			

*V*: variants (alleles A4, Av1-Av5).

**Table 4:** Association study on indel locus of *GIGYF2* by genotypic and allelic stratification.

*GIGYF2*	PD Case (%)	Control (%)	*χ*2	*P*	OR (95% CI)	Power
*X*	396 (0.569)	318 (0.489)	8.579	0.003	1.378 (1.112, 1.708)	0.833
*5*	300 (0.431)	332 (0.511)				
*X/X*	115 (0.330)	86 (0.265)	8.916	0.012	-	0.767
*5/X*	166 (0.477)	146 (0.449)				
*5/5*	67 (0.193)	93 (0.287)				
*5/X+X/X*	281 (0.807)	232 (0.663)	8.129	0.004	1.681 (1.174, 2.407)	0.814
*5/5*	67 (0.193)	93 (0.287)				

*X*: alleles *6*, *7*, A4 and Av1-Av5. *P* < 0.05/2 represents statistics significant for multiple testing.

### Differences in the genetic distribution of PD-associated indels according to gender

We performed gender stratification to better understand the association between the three indels and PD. As expected, no association was found between the indels in the *ACE* and *DJ-1* genes and PD in the male or female datasets when studied separately (S2 Table). Although statistical significance was found in males, but not females, between patients and controls for the *GIGYF2* indel (Table 5), no difference between males and females was found in either PD cases or controls (Table 6).

**Table 5:** Association study on indel locus of *GIGYF2* by gender stratification.

*GIGYF2*	Male					Female				
	PD Case	Control	*P*	OR (95% CI)	Power	PD Case	Control	*P*	OR (95% CI)	Power
*X*	210	217	0.022	1.383 (1.047, 1.829)	0.626	186	101	0.061	1.394 (0.985, 1.973)	0.467
*5*	156	223				144	109			
*X/X*	60	59	0.051	-	0.580	55	27	0.185	-	0.357
*5/X*	90	99				76	47			
*5/5*	33	62				34	31			
*5/X+X/X*	150	158	0.017	1.784 (1.106, 2.876)	0.666	131	74	0.095	1.614 (0.918, 2.837)	0.386
*5/5*	33	62				34	31			

*X*: alleles *6*, *7*, A4 and Av1-Av5.

**Table 6:** Comparison of *GIGYF2* indel locus between male and female.

*GIGYF2*	PD Case					Control				
	Male	Female	*χ*2	*P*	OR (95% CI)	Male	Female	*χ*2	*P*	OR (95% CI)
*X*	210	186	0.073	0.787	1.042 (0.772, 1.407)	217	101	0.085	0.771	1.050 (0.756, 1.459)
*5*	156	144				223	109			
*X/X*	60	55	0.483	0.785	-	59	27	0.078	0.962	-
*5/X*	90	76				99	47			
*5/5*	33	34				62	31			
*5/X+X/X*	150	131	0.370	0.543	1.180 (0.682, 2.011)	158	74	0.062	0.803	1.068 (0.640, 1.781)
*5/5*	33	34				62	31			

*X*: alleles *6*, *7*, A4 and Av1-Av5.

## Discussion

In the current study, we selected three different indels to evaluate their role as predisposing factors for PD. Our results showed no significant differences in the allelic frequencies of indels in the *ACE* and *DJ-1* genes between PD patients and controls. However, an association between the *GIGYF2* indel polymorphism and PD was found in the population. With regard to the genetic factors associated with complex diseases or phenotypes, comprehensive understanding for different variations may be needed [28]. Single nonfunctional variants as susceptible locus, are often related to a disease or phenotype, which implied that these variants are actually in strong linkage with causal mutations. Therefore, results based on single variant in related loci can lead to incorrect explanation for complex phenotypes. Some studies have demonstrated loci with multiple variants in complete linkage disequilibrium with the indel polymorphisms [29,30]. It is noteworthy that the causal mutation may be better captured by haplotype-based methods [31,32]. Based on these issues, it is plausible that the indel polymorphisms in this study, whether they are susceptible for PD or not, must be considered for haplotype investigations to make clear relationship between indels and the true risk variants.

The indel polymorphism in the *ACE* gene is a transposon insertion in intron 16. These mobile genetic elements produce a number of small indel variations in humans [33], i.e., the *Alu* transposon in *ACE*. It was originally reported that the ACE activity levels in *D/D* carriers were approximately twice those found in *I/I* genotype individuals [34]. Therefore, this indel polymorphism was used as a marker for studying associations with pathophysiological conditions, including neurodegenerative diseases, such as PD [10]. Moreover, the *ACE* indel polymorphism in the Australian Caucasian population was not associated with PD in the first association study [35]. No statistically significant differences were found in alleles or genotypes for *ACE* indel polymorphism during investigations of Caucasian populations in Greece, Italy, and Columbia [36–38]. However, one report showed that the frequency of the homozygote *D/D* genotype in the *ACE* gene was considerably increased in patients with PD compared to controls in a Taiwanese population, despite there being no significant difference in allelic frequency [12]. In the current study, the *I* vs. *D* genetic model in the *ACE* indel locus did not result in a statistically significant association. Certain other reports from the literature have implied that different ages and ethnicities might be an explanation for the disparate results. On the other hand, power analysis was performed after the experiment was finished and was lower than 0.80, which suggest that more samples were needed to estimate the distributions. Although this study reported a negative result, it was the first to investigate the *ACE* indel polymorphism’s influence on PD patients from the Chinese mainland. Further studies are needed to analyze the linkage disequilibrium between indels and multiple loci in this region of *ACE* and to evaluate the role of this indel in the incidence of Parkinson’s disease in populations from other areas of the Chinese mainland.

Given that variants within the promoter region of both ɑ-synuclein and parkin are associated with increased risk for sporadic PD [2,27], the *DJ-1* indel polymorphism was of particular interest. The 18-bp indel polymorphism in the *DJ-1* gene can be classified as a random sequence type. Hague *et al*. was the first to identify several non-coding variants in *DJ-1*, including the indel in the *DJ-1* gene located in the promoter region and near the transcription regulatory SP1 site [39]. This polymorphism is hypothesized to affect gene expression due to its proximity to the transcriptional regulatory SP1 site. Furthermore, this indel may be involved in DJ-1 protein expression, subsequently influencing neuronal response to oxidative stress. Its association with genetic susceptibility to PD has been previously reported in several different populations although some association studies have indicated mixed results. The *DJ-1* indel polymorphism did not confer a risk for PD in studies conducted in Finland and Great Britain [40,41]. Nevertheless, the deletion allele of the indel locus was found to be a risk factor for PD in Italian and Indian case-control studies [20,21]. In addition, two investigations in southern Africa and west China, determined that the deletion allele occurred at very low frequencies in these populations and was, therefore, unlikely to play a significant role in PD [42,43]. In the present study, we also did not find that the *DJ-1* indel was associated with PD in our population from northern China. Because the statistical power was low, the efficiency may increase for this polymorphism with a larger sample size.

In this study, we identified the *X* allele the *5/X+X/X* genotype of the *GIGYF2* gene as risk factors that enhanced the risk of PD (P < 0.05). The power was approximately 0.80. Our results are the first to suggest that indels in the *GIGYF2* gene may be associated with PD in a northern Chinese population. The GIGYF2 protein is involved in insulin-like growth factor (IGF) and insulin signaling in the central nervous system [22,23]. Moreover, the *GIGYF2* gene has been reported to be a susceptibility gene responsible for PARK11-linked PD [24]. Given the current data and previously reported findings, we conclude that the architecture of the indel locus most likely represents a repeat extension of an irregular 3-bp multiple model. The *GIGYF2* indel was first identified when screening for *GIGYF2* gene mutations in Italian and French patients with familial PD [24]. Until now, only 3-bp insertion or deletion alleles in exon 29 of the *GIGYF2* gene have been detected. Although the indel locus might not play a pathogenic role in PD, the existence of multiple alleles and genotypes in the poly Q region is still meaningful because polyglutamine expansions can cause neurodegenerative disorders [44,45]. Lautier *et al*. identified seven different alleles in the indel locus, but did not confirm a causal role in familial PD [24]. Studies in Asian (Taiwanese and Singapore) [46] (Japanese) [47], Portuguese and North American [48], and Italian cohorts [49] suggest that the indel in exon 29 of *GIGYF2* is not strongly related to the development of PD based on mutational analysis, although different alleles were identified. For the association of indel polymorphism with PD, it appears to be comparatively lower because the 3-bp indel locus in exon 29 displays an irregular trait. Nichols *et al*. identified seven alleles in Caucasian population in North America [50]. Compared with the wild type (allele *6* in our study), an allele with one Q deleted (allele *5* in our study) was present at a higher frequency, while other alleles were present at a lower frequency. However, there was no evidence that any of these *GIGYF2* indels increased the risk for PD. Moreover, Cao *et al*. analyzed the DNA sequence surrounding exon 29 of *GIGYF2* and found that two alleles with one deleted (allele *5*) and inserted Q (allele *7*) were present at higher frequencies. The allele with an inserted Q was more prevalent in the control group, which suggested it was more likely to be a benign variant [51]. In the current study, we combined DNA sequencing with allele length analysis and found eight different alleles in northern China. First, we applied allele length polymorphism analysis to evaluate the association of the *GIGYF2* indel locus with PD. The results of this study indicated that: (1) all alleles are 3-bp fold different and alleles *5*, *6*, and *7* are present at higher frequencies, as reported by Cao *et al*. in Shanghai, China. No deviation from Hardy–Weinberg equilibrium was observed based on genotype distribution, which suggests that allele length analysis can be used in association studies. (2) Although the distributions of genotypic frequency between both groups indicated that the indel is unlikely to influence PD development in northern Han Chinese, allele *X* conveys an increased risk in both the allelic *X* vs. *5* and *5/X +X/X* vs. *5/5* genetic models. These results suggest that other genetic groups and districts can be comparaed using these models. (3) The uncommon alleles found in this study seem to present preferentially in PD patients, which suggests that the complexity and diversity in exon 29 of the *GIGYF2* gene may influence its function and lead to cellular degeneration.

Subsequently, we conducted a gender stratification analysis of the relationship between the frequency distributions of the three indel polymorphisms in different models and susceptibility to PD. Many mutations and polymorphisms leading to gene deficiency or linkage association with diseases exhibit gender-linked differences [52,53]. Simunovic *et al*. found differential gene expression in both males and females with distinct PD-association patterns and described a bias of the male gender towards PD at the molecular level [54]. Furthermore, a common mutation associated with PD, G2019S in the *LRRK2* gene, was found to have a relatively greater genetic load in women with PD among individuals of Jewish descent [55]. Interestingly, rs12817488 in the *CCDC62* gene was associated with PD risk, but the results were unclear after stratification by gender. Yu *et al*. found that a significant association only existed in males [56], while Liu *et al*. only found this association in a female population [57]. In summary, several aspects may contribute to the different results between the two studies, including age of onset, EOPD/LOPD ratio, sex ratio, and MAF [58]. Our previous study also observed a genetic female distribution bias in the mitochondrial genome of PD patients in northern China [59]. Taken together, the above data indicate that the genetic effect might be specific to either males or females. In the present study, we continued to analyze gender-relevant traits associated with several genetic markers specific for PD in northern China. However, no sex dependent effects on PD were found for these three indel loci. Comparing previous data with present data, the genetic markers that we describe in this study are associated with PD in northern China and show complex characteristics in both overall and gender stratification, suggesting that it is vital to accurately assess the genetic architecture of PD patient populations as the number of genetic markers increases.

## Conclusions

Overall, our result revealed that genotype *5/5* in the *GIGYF2* gene may be a protective factor for PD patients in northern China, but no differences were found in either *ACE* or *DJ-1*. Although there was the statistical significance comparing with frequencies of allele *5*, the allele X is a composite class of alleles. Therefore, these alleles may be associated markers and can be linked to the causative mutation.

## Supporting information

S1 Table The genotype frequency distribution and association analysis of the indel loci of the *ACE* and *DJ-1* genes. (DOC)

S2 Table Allele association analysis after gender stratification of the indel loci in the *ACE*, *DJ-1*, and *GIGYF2* genes. a: variant allele in *ACE*; b: variant allele in *DJ-1*; *V*: variants (alleles A4, Av1-Av5).
(DOC)

S3 Table Association study of the indel in the *GIGYF2* gene stratified by allele. *X*: alleles *5*, *7*, A4, and Av1-Av5.
(DOC)

S4 Table Association study of the indel in the *GIGYF2* gene stratified by allele. *X*: alleles *5*, *6*, A4, and Av1-Av5.
(DOC)
