# Supplementary material for: Genetic analysis of indel markers in three loci associated with Parkinson's disease
Source: PLoS One. 2017 Sep 5;12(9):e0184269. doi: 10.1371/journal.pone.0184269 (PMC5584932; doi:10.1371/journal.pone.0184269)
Supplement: S1 Table — (DOC) [file pone.0184269.s001.doc]

**S1 Table. The genotype frequency distribution and association analysis of the indel loci of the *ACE* and *DJ-1* genes.**

| **Variables** | **PD Case (%)** | **Control (%)** | ***χ*2** | ***P*** |
| --- | --- | --- | --- | --- |
| *ACE* |  |  |  |  |
| *D/D* | 35 (10) | 50 (15) | 5.362 | 0.068 |
| *I/D* | 168 (48) | 136 (42) |  |  |
| *I/I* | 145 (42) | 139 (43) |  |  |
|  |  |  |  |  |
| *DJ-1* |  |  |  |  |
| *I/I* | 285 (82) | 268 (82) | 0.809 | 0.667 |
| *I/D* | 60 (17) | 52 (16) |  |  |
| *D/D* | 3 (1) | 5 (2) |  |  |
