# Supplementary material for: Genetic analysis of indel markers in three loci associated with Parkinson's disease
Source: PLoS One. 2017 Sep 5;12(9):e0184269. doi: 10.1371/journal.pone.0184269 (PMC5584932; doi:10.1371/journal.pone.0184269)
Supplement: S2 Table — a: variant allele in ACE; b: variant allele in DJ-1; V: variants (alleles A4, Av1-Av5). (DOC) [file pone.0184269.s002.doc]

**S2 Table. Allele association analysis after gender stratification of the indel loci in the *ACE*, *DJ-1* and *GIGYF2* genes.**

| **Variables** | **PD Case** | **Control** | ***P*** | **OR (95% CI)** | **Power** |
| --- | --- | --- | --- | --- | --- |
| *ACE* |  |  |  |  |  |
| Male |  |  |  |  |  |
| *I^a^* | 235 | 275 | 0.617 | 1.076 (0.807, 1.436) | 0.079 |
| *D* | 131 | 165 |  |  |  |
| Female |  |  |  |  |  |
| *I* | 223 | 139 | 0.739 | 1.065 (0.737, 1.537) | 0.063 |
| *D* | 107 | 71 |  |  |  |
|  | | | | |  |
| *DJ-1* |  |  |  |  |  |
| Male |  |  |  |  |  |
| *D^b^* | 334 | 391 | 0.261 | 1.308 (0.819, 2.090) | 0.203 |
| *I* | 32 | 49 |  |  |  |
| Female |  |  |  |  |  |
| *D* | 34 | 13 | 0.098 | 1.741 (0.896, 3.381) | 0.379 |
| *I* | 296 | 197 |  |  |  |
|  | | | | |  |
| *GIGYF2* |  |  |  |  |  |
| Male |  |  |  |  |  |
| *5* | 156 | 223 | 0.102 | - | 0.534 |
| *6* | 102 | 114 |  |  |  |
| *7* | 102 | 99 |  |  |  |
| *V* | 6 | 4 |  |  |  |
| Female |  |  |  |  |  |
| *5* | 144 | 109 | 0.163 | - | 0.450 |
| *6* | 100 | 56 |  |  |  |
| *7* | 83 | 45 |  |  |  |
| *V* | 3 | 0 |  |  |  |

a: variant allele in *ACE*; b: variant allele in *DJ-1*; *V*: variants (alleles A4, Av1-Av5)*.*
