# Supplementary material for: Genetic analysis of indel markers in three loci associated with Parkinson's disease
Source: PLoS One. 2017 Sep 5;12(9):e0184269. doi: 10.1371/journal.pone.0184269 (PMC5584932; doi:10.1371/journal.pone.0184269)
Supplement: S3 Table — X: alleles 5, 7, A4, and Av1-Av5. (DOC) [file pone.0184269.s003.doc]

**S3 Table.** **Association study of the indel in the *GIGYF2* gene stratified by allele.**

| ***GIGYF2*** | **PD Case (%)** | **Control (%)** | ***P*** | **OR (95% CI)** | **Power** |
| --- | --- | --- | --- | --- | --- |
| *X* | 494 (0.710) | 480 (0.738) | 0.240 | 0.866 (0.682, 1.101) | 0.217 |
| *6* | 202 (0.290) | 170 (0.262) |  |  |  |
| *X: alleles 5, 7, A4, and Av1-Av5.* | | | | | |
