# Supplementary material for: Genetic analysis of indel markers in three loci associated with Parkinson's disease
Source: PLoS One. 2017 Sep 5;12(9):e0184269. doi: 10.1371/journal.pone.0184269 (PMC5584932; doi:10.1371/journal.pone.0184269)
Supplement: S4 Table — X: alleles 5, 6, A4, and Av1-Av5. (DOC) [file pone.0184269.s004.doc]

**S4 Table. Association study of the indel in the *GIGYF2* gene stratified by allele.**

| ***GIGYF2*** | **PD Case (%)** | **Control (%)** | ***P*** | **OR (95% CI)** | **Power** |
| --- | --- | --- | --- | --- | --- |
| *X* | 511 (0.734) | 506 (0.778) | 0.059 | 0.786 (0.612, 1.009) | 0.472 |
| *7* | 185 (0.266) | 144 (0.222) |  |  |  |

*X*: alleles *5*, *6*, A4 and Av1-Av5.
